# Identification of transcription factors potential related to brown planthopper resistance in rice via microarray expression profiling

**DOI:** 10.1186/1471-2164-13-687

**Published:** 2012-12-10

**Authors:** Yubing Wang, Huimin Guo, Haichao Li, Hao Zhang, Xuexia Miao

**Affiliations:** 1 Key Laboratory of Insect Developmental and Evolutionary Biology, Institute of Plant Physiology and Ecology, Shanghai Institutes for Biological Sciences, Chinese Academy of Sciences, Shanghai 200032, P.R. China; 2 Graduate School of Chinese Academy of Sciences, Shanghai, 200032, P.R. China

**Keywords:** Transcription factor, *Oryza sativa L*, *Nilaparvata lugens* Stål, Microarray

## Abstract

**Background:**

Brown planthopper (BPH), *Nilaparvata lugens* Stål, is one of the most destructive insect pests of rice. The molecular responses of plants to sucking insects resemble responses to pathogen infection. However, the molecular mechanism of BPH-resistance in rice remains unclear. Transcription factors (TF) are up-stream regulators of various genes that bind to specific DNA sequences, thereby controlling the transcription from DNA to mRNA. They are key regulators for transcriptional expression in biological processes, and are probably involved in the BPH-induced pathways in resistant rice varieties.

**Results:**

We conducted a microarray experiment to analyze TF genes related to BPH resistance in a Sri Lankan rice cultivar, Rathu Heenati (RHT). We compared the expression profiles of TF genes in RHT with those of the susceptible rice cultivar Taichun Native 1 (TN1). We detected 2038 TF genes showing differential expression signals between the two rice varieties. Of these, 442 TF genes were probably related to BPH-induced resistance in RHT and TN1, and 229 may be related to constitutive resistance only in RHT. These genes showed a fold change (FC) of more than 2.0 (*P*<0.05). Among the 442 TF genes related to BPH-induced resistance, most of them were readily induced in TN1 than in RHT by BPH feeding, for instance, 154 TF genes were up-regulated in TN1, but only 31 TF genes were up-regulated in RHT at 24 hours after BPH infestation; 2–4 times more TF genes were induced in TN1 than in RHT by BPH. At an FC threshold of >10, there were 37 induced TF genes and 26 constitutive resistance TF genes. Of these, 13 were probably involved in BPH-induced resistance, and 8 in constitutive resistance to BPH in RHT.

**Conclusions:**

We explored the molecular mechanism of resistance to BPH in rice by comparing expressions of TF genes between RHT and TN1. We speculate that the level of gene repression, especially for early TF genes, plays an important role in the defense response. The fundamental point of the resistance strategy is that plants protect themselves by reducing their metabolic level to inhibit feeding by BPH and prevent damage from water and nutrient loss. We have selected 21 TF genes related to BPH resistance for further analyses to understand the molecular responses to BPH feeding in rice.

## Background

Rice, *Oryza sativa L*., is the staple food of more than three billion people in Asia. In natural environments, rice plants are often attacked by microbial pathogens and insect pests. One of the most destructive insect pests of rice is the brown planthopper (BPH), *Nilaparvata lugens* Stål. BPH use their stylets to probe intercellularly through epidermal and mesophyll cell layers until they reach the phloem sieve element to extract phloem sap as their food [1]. This differs from the feeding pattern of chewing insects. Their feeding activity extracts the phloem sap of rice and causes damage known as ‘hopper burn’, which can be lethal to rice plants attacked by large populations of the insect. Their feeding also transmits certain rice viruses such as ragged stunt virus and grassy stunt virus [2]. In recent years, BPH infestations have devastated many rice crops in Asia [3]. Therefore, the rice resistance against BPH is very important to rice production. Generally, according to the gene’s expression level whether can be affected by BPH attack, the rice resistance were divided into induced resistance and constitutive resistance. The “induced resistance” refers to induced changes in preference, performance, or reproductive success of the attacker [4]. Most of plants will produce induced resistance reaction once they were attacked by herbivore insects. While in contrast, constitutive plant resistance refers to the constant level of resistance in a plant, regardless of herbivore attack. Most prominent resistance factor, for example the morphological features and the chemical composition of the plant, have been recognized as constitutive resistance characters [5].

Few studies have been conducted on the molecular responses of plants to sucking insects. Most studies on plant defense responses to phloem-feeding insects have focused on aphids and whiteflies [6,7]. In the interaction between rice and BPH, gene expression is controlled after activation of salicylic acid (SA)-dependent and jasmonic acid (JA)/ ethylene (ET)-dependent signaling pathways [8,9]; this was thought to be a response to pathogen infection [10]. Analyses of physiological responses and gene expression profiles in rice showed that the genes significantly induced or repressed by BPH infestation were involved in several different pathways, including cellular transport, signal transduction, metabolism, macromolecular degradation, and plant defenses [9,11,12]. Although differential expression profiles related to BPH infestation have been analyzed by several groups, the molecular mechanism of BPH resistance in rice is remains unclear.

Previously, we conducted a series of microarray analyses to find potential BPH resistance genes in a Sri Lankan rice cultivar, Rathu Heenati (RHT). We compared its gene expression profiles under BPH stress with those of the susceptible cultivar Taichun Native 1 (TN1), which was used as the negative control [13]. Using this screening strategy, we identified many transcription factor (TF) genes related to BPH resistance. The products of these genes play important regulatory roles in many resistance-related pathways. TFs are proteins that can bind to specific DNA sequences, thereby controlling the transcription from DNA to mRNA [14,15]. They are key regulators of transcriptional expression in many biological processes [16]. Previous studies also indicated that TF genes directly or indirectly regulate the plant defense response [17-20]. In the model plant *Arabidopsis*, bHLH and WRKY gene families have been implicated in *R* gene-mediated resistance against aphids [18]. The *Arabidopsis* TF AtWRKY70 modulates the cross-talk between the salicylic acid and jasmonic acid signaling pathways, which play important roles in plant defense against pathogens [21-23]. Over-expression of a rice TF gene, *WRKY89,* increased plant resistance to white-backed planthopper, a phloem-feeding insect [24]. *OsERF3* is another rice TF gene that belongs to the AP2/EREBP family. It positively regulates TrypPI activity and plays a role in the resistance of rice to the chewing herbivore, rice striped stem borer. It also appears to be a negative regulator of resistance against BPH [8]. TF genes that contain a zinc finger motif have been implicated in the regulation of plant tolerance to biotic or abiotic stresses [25-28]. Other TF families, including MYB [29,30], NAC domain-containing [31-34], and bZIP families [35-38], also have important roles in plant-defense response pathways. DNA microarray technology provides a high-throughput method to measure expression levels of thousands of genes simultaneously. The technique is a powerful tool for global analysis of altered gene expression in plants under different conditions [39-41]. The results of previous studies suggest that it is feasible and practical to use microarrays to measure the expression levels of TF genes in rice plants infested by BPH.

In this study, we analyzed the expression profiles of 2720 TF genes in an Affymetrix rice genome array at two time points during the plant-BPH interaction. Analyses of differential expression profiles between susceptible (TN1) and resistant (RHT) rice cultivars allowed us to identify TF genes that showed significant changes in expression levels after BPH infestation. We identified 37 induced and 26 constitutive TF genes related to BPH resistance. Of these, 21 TF genes were further analyzed and identified as those that were most likely to be related to BPH resistance in RHT. We speculate about the molecular mechanisms of resistance to BPH in RHT based on the expression patterns of TF genes after BPH feeding.

## Methods

### Plant and insect materials

We used the BPH-susceptible rice cultivar Taichung Native 1 (TN1) and the BPH-resistant cultivar Rathu Heenati (RHT) [42]. Pre-germinated seeds were sown in a plastic tray (60×45×45 cm) and were grown under a 14-h light/10-h dark photoperiod at 28/22°C. The TN1 cultivar was used to raise the BPH populations.

The BPH population was obtained from the China National Rice Research Institute, Fuyang, Zhejiang. The BPH population was reared in a greenhouse under the same temperature and light regime described above. We used second or third instar nymphs of BPH for infestation experiments, and the fourth or fifth instar nymphs for the host-plant choice experiment.

### BPH infestation and sample collection

The second or third instar nymphs were transferred to 6-week-old seedlings (10 BPH nymphs per plant) in a box covered with nylon mesh. Stems of the rice plant infected by BPH were collected as samples for microarray and qPCR at 0, 2, 4, 8, 12, 18, 24, or 36 h after BPH infestation. The stems were transferred to liquid nitrogen immediately and then stored at −70°C.

The herbivore host-plant choice experiment was performed in plastic pots with two plants each. To determine the colonization preferences of BPH, the two plants were covered with a plastic cage for each experiment (diameter 15 cm, height 50 cm), and 20 nymphs of 4~5 instar BPH were introduced into the cage. The BPH nymphs on each plant were counted at 2, 4, 6, 8, 10, 12, 24, 36, 48, and 72 h after release. This experiment had five replicates.

### RNA extraction, Affymetrix microarray hybridization, and data normalization

Total RNA was extracted using the QIAGEN RNA Extraction kit (QIAGEN, Hilden, Germany) according to the manufacturer’s instructions. The RNA samples were purified with the QIAGEN RNAeasy kit (QIAGEN, Hilden, Germany), and an Agilent 2100 Bioanalyzer was used to monitor sample quality. Only RNA samples from the 0, 8, and 24 h time-points were used for Affymetrix microarray hybridizations.

The Shanghai Bio Corporation carried out Affymetrix rice genome microarray hybridizations. RNA target preparation and microarray hybridization were performed with the GeneChip® 3’ IVT Express kit (Affymetrix, Cleveland, USA) and GeneChip® Hybridization, Wash, and Stain kit (Affymetrix, Cleveland, USA), strictly following the manufacturer’s instructions. The signal intensity for each probe set on the GeneChip microarray was detected with a GeneChip® Scanner 3000 (Affymetrix, Cleveland, USA), and the raw signal value for each probe set was analyzed with GeneChip operating software (GCOS; Affymetrix, Cleveland, USA). The original data has already submitted to GEO, and the GEO record is “GSE29967 - Expression data from rice after brown planthopper attack”.

For microarray analyses, we included six treatments of RHT and TN1 before and after BPH infestation with three biological repeats for each treatment. We conducted quintile normalization for all microarrays using MAS 5.0 to standardize the distribution of probe intensities for each array in a set of arrays. For quality control of samples, we compared all sample expression files using principal component analysis (PCA) to ensure that all samples representing the same experimental conditions were similar to each other. Primary screening was performed with Genespring GX 11.5 (Agilent Technologies, Santa Clara, CA) using one-way ANOVA and a Benjamini Hochberg false discovery rate threshold of less than 0.05. The expression values were compared pair-wise with the fold-change tool and a Student’s t-test was performed during this step to obtain the P-value for each probe set.

### Transcription factor screening and microarray probe set filters

The rice transcription factors analyzed in this experiment were described in the transcription factor database [43]. According to the annotation of Affymetrix genome microarray (release 30), we screened for TF genes that were differentially induced or repressed after BPH infection in RHT and TN1 with a fold change (FC) of >2.0 and a *P*-value of <0.05. The results were shown as a Venn diagram ( http://bioinformatics.psb.ugent.be/webtools/Venn/). Further probe filtering for TF genes that were significantly induced by BPH or constitutively expressed in the resistant cultivar RHT was performed with the fold-change tool in Genespring GX 11.5.

### Quantitative reverse-transcription PCR (qPCR) analyses

Purified RNA samples were treated with RNase-free DNase (NEB, Ipswich, USA) at 37°C for 1 h. Reverse transcription was performed with the RevertAid™ First Strand cDNA Synthesis kit (Fermentas, Boston, USA) using Oligo(dT)_18_ primers. The reactions were incubated at 42°C for 60 min and 70°C for 5 min, chilled on ice for 5 min, and the cDNA was stored at −20°C until use. qPCR analyses were performed with Maxima™ SYBR Green qPCR Master Mix (2×) (Fermentas, Boston, USA) in 25-μl reaction mixtures following the manufacturer’s instructions. The actin gene was used as an internal control to normalize Ct values obtained for each gene. Data analysis was performed according to the methods described by Livaka and Schmittgen [44]. Primer pairs are given in Online Resources (Additional file 1); these were designed using the PCR primer design tool primer3 ( http://frodo.wi.mit.edu) according to the probe set target sequences accessible on the Affymetrix website http://www.affymetrix.com/analysis/index.affx).

## Results

### RHT has an efficient resistant mechanism to brown planthopper

**Figure 1:**
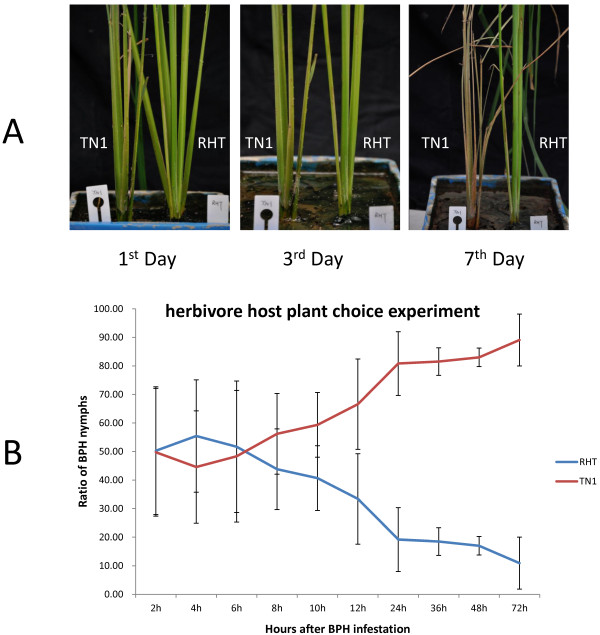
**Brown planthopper (BPH) feeding and selection test on rice varieties RHT (resistant) and TN1 (susceptible).A**, Phenotypes of RHT and TN1 on days 1, 3, and 7 of BPH infestation. **B**, BPH host selection experiment between rice varieties RHT and TN1 (72 h).

The Sri Lankan rice variety RHT contains a resistance gene, *Bph3*, and shows resistance to all four biotypes of BPH [42]. This variety has an efficient resistance mechanism to BPH [42]. A previous study showed a low survival rate of BPH populations on RHT [1]. Another study on BPH showed that up to 80% of their time was spent on non-feeding behaviors, and they could not draw phloem sap from RHT [45]. When RHT and TN1 were exposed to BPH, RHT was less affected and survived longer than TN1. By 7 days of BPH infestation, TN1 plants were dead and dry, whereas RHT plants were still alive (Figure 1A). The herbivore host-plant choice experiment showed that BPH nymphs chose host plants randomly during the first 6–8 h, but showed a preference for TN1 from 8 h after BPH infestation (Figure 1B). These findings indicated that RHT has an efficient resistance mechanism to BPH.

### Transcription factor expression profiles in susceptible or resistant rice varieties

We compared a series of Affymetrix rice genome arrays to measure the expression profiles of TF genes in two rice varieties before and after BPH infestation. According to the descriptions in the transcription factors database [43], we extracted a total of 2720 probes from the array that were annotated as TFs. In this study, the probes revealed 2038 TF genes (74.9%) that were expressed at detectable levels after normalization. These could be subdivided into 79 gene families (Additional file 2). All of these TF genes were analyzed by pair-wise comparisons between resistant and susceptible varieties before and after BPH infestation using Genespring GX 11.5 software (Agilent Technologies). After comparison, of the 2038 TF genes with detectable expressions, 442 were identified as being related to BPH-induced resistance and 229 TF genes were related to constitutive resistance in RHT (fold change >2, *P*<0.05) (Additional files 3 &4). Those TF genes that were related to induced resistance were up-regulated or down-regulated by BPH attack. Those that were related to constitutive resistance were up-regulated or down-regulated in RHT compared with their respective expressions in TN1 at the same time points. These genes are considered as being specific to the resistant rice variety, RHT.

### Potential transcription factor genes associate with BPH-induced resistance

**Figure 2:**
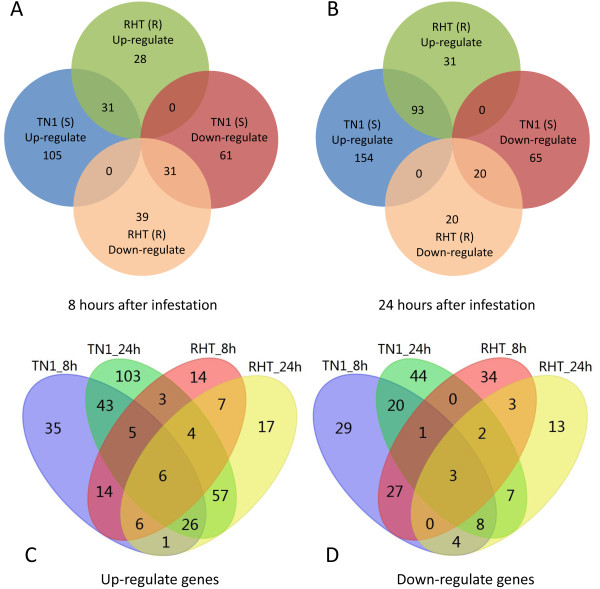
**Analysis of 442 TF genes showing differential expression between resistant (RHT) and susceptible (TN1) rice varieties during BPH infestation.A**, Up- or down-regulated TF genes in RHT or TN1 at 8 h after BPH infestation. **B**, Up- or down-regulated TF genes in RHT or TN1 at 24 h after BPH infestation. **C**, Up-regulated TF genes in RHT and TN1 at 8 and 24 h after BPH infestation. **D**, Down-regulated TF genes in RHT and TN1 at 8 and 24 h after BPH infestation.

As described above, there were 442 genes that were probably related to BPH-induced resistance. These showed differential expressions between BPH-infested and non-infested plants. Further analysis showed that in the resistant rice variety RHT, 28 TF genes were specifically up-regulated and 39 genes were specifically down-regulated at 8 h after BPH infestation (Figure 2A); at 24 h after BPH infestation, 31 genes were specifically up-regulated and 20 genes were specifically down-regulated (Figure 2B). However, at the same time points, there were 2–3 times more TF genes that were up- or down-regulated by BPH attack in the susceptible rice variety TN1 (Figures 2A &2B).

As shown in Figures 2A and 2B, there were 105 up-regulated TF genes in TN1 at 8 h after infestation and 154 at 24 h after infestation. There were 61 and 65 down-regulated genes at 8 and 24 h after infestation, respectively. In contrast, in the resistant variety RHT, there were more down-regulated than up-regulated genes after BPH infestation (Figures 2A).

The up- or down-regulated TF genes at two time points were further analyzed (Figures 2C &2D). There were more TF genes up- or down-regulated in TN1 than in RHT after BPH attack. In other words, expressions of more TF genes were induced in the susceptible rice variety TN1 than in the resistant rice variety RHT. However, only a few genes were commonly expressed in the two rice varieties at different time points after BPH attack (Figures 2C &2D).

### Main families of TF genes related to BPH resistance

**Figure 3:**
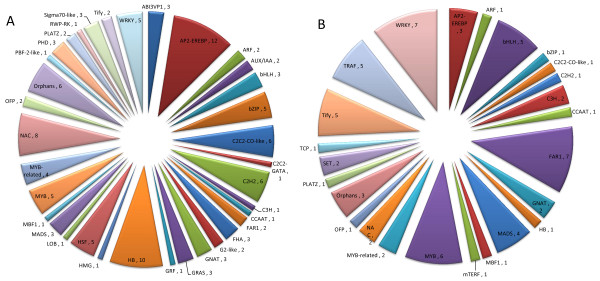
**Classification of TF gene families showing differential expression with fold-change values of >5 (*P*<0.05).A**, Gene families of 119 induced TF genes (fold change >5, *P*<0.05). **B**, Gene families of 66 constitutive TF genes (fold change >5, *P*<0.05).

A fold change of >5 was considered to indicate significantly differentially expressed TF genes. There were 119 TF genes related to induced resistance and 66 related to constitutive resistance with fold change values of >5 (Additional files 5 &6). The 119 TF genes related to induced resistance belonged to 35 TF gene families, mainly the WRKY, AP2-EREBP, HB, MYB, and NAC families (Figure 3A). The 66 TF genes related to constitutive resistance belonged to 25 families, mainly the WRKY, bHLH, MYB, and TRAF families (Figure 3B).

Further analyses indicated that there were significant differences in the expressions of zinc-finger TF family members between induced and constitutive TF genes. For instance, C2C2-CO like and C2H2 were the main zinc finger TF genes among the genes related to BPH-induced resistance, whereas Tify and TRAF were the main zinc finger TF genes among those related to constitutive resistance (Figures 3A &3B).

### Further analysis of TF genes related to BPH-induced resistance

To further identify TF genes that were induced by BPH and associated with BPH resistance, we screened 37 genes with FC values of >10 (*P*<0.05) (Table 1). According to the FC value after BPH attack and the resistance of the two rice varieties to BPH, the induced TF genes were divided into three categories: (1) Those that were strongly up- or down-regulated in TN1, but unaffected or barely affected in RHT (FC<5). These TF genes are likely to be sensitive to a biological stress, but are unlikely to participate in BPH-resistance (Table 1, lines 1–14). (2) Those that were strongly up- or down-regulated at 8 h after BPH infestation in the two rice varieties, but showed expression levels similar to those before BPH attack thereafter. These genes probably participate in the early stages of the biological stress response (Table 1, lines 15–24). (3) Those that were up- or down-regulated in both rice varieties, or strongly down-regulated only in RHT. These TF genes were likely to be involved in BPH-inducible resistance, and included the remaining 13 TF genes (Table 1, lines 25–37).

**Table 1:** **TF genes related to BPH-induced resistance (fold change >10; *P*<0.05)**

**No**	**Family**	**Gene ID**	**T**_**8**_**/T**_**0**_		**T**_**24**_**/T**_**0**_		**R**_**8**_**/R**_**0**_		**R**_**24**_**/R**_**0**_	
			**FC**	**RP**	**FC**	**RP**	**FC**	**RP**	**FC**	**RP**
1	ABI3VP1	Os03g0164300	10.14	U	10.28	U	1.50	U	4.31	U
2	AP2-EREBP	Os01g0224100	1.91	D	12.40	D	2.66	D	1.53	D
3	AP2-EREBP	Os03g0860100	19.22	U	8.30	U	2.13	U	2.08	U
4	bZIP	Os03g0336200	4.37	U	10.25	U	1.55	U	3.08	U
5	C2C2-CO-like	Os09g0240200	29.47	D	1.22	D	3.57	D	1.83	U
6	C2H2	Os03g0437200	10.14	U	3.27	U	2.00	U	1.67	U
7	FAR1	Os08g0389800	7.46	U	11.87	U	1.36	U	1.36	D
8	FHA	Os12g0124000	14.24	D	1.23	D	3.91	D	1.38	D
9	GRAS	Os01g0948200	14.58	U	6.82	U	1.30	D	2.28	U
10	HB	AK064665	8.88	D	14.77	D	2.18	D	3.32	D
11	NAC	Os02g0214500	10.52	U	6.62	U	1.27	D	2.11	D
12	RWP-RK	Os09g0549450	2.69	D	10.01	D	1.39	D	2.83	D
13	Tify	Os03g0180800	10.45	U	1.51	U	4.29	U	3.75	U
14	WRKY	Os03g0335200	6.85	U	10.14	U	2.13	U	2.02	U
15	AP2-EREBP	Os07g0410700	5.35	U	1.73	U	10.02	U	3.12	U
16	C2C2-CO-like	Os06g0298200	34.79	U	1.41	U	23.97	U	1.02	U
17	C2C2-CO-like	Os09g0509700	11.88	U	2.39	U	5.49	U	2.53	U
18	G2-like	Os01g0971800	1789.73	U	1.71	U	804.09	U	2.39	U
19	HSF	Os01g0733200	32.45	U	2.64	U	45.58	U	3.06	U
20	MBF1	Os06g0592500	12.14	D	1.58	D	5.60	D	2.77	D
21	MYB-related	Os08g0157600	45.27	D	2.13	U	47.13	D	1.70	U
22	Orphans	Os04g0493000	5.34	D	2.08	U	12.51	D	1.54	U
23	Sigma70-like	Os05g0586600	24.58	D	2.50	U	92.84	D	1.87	U
24	Sigma70-like	Os05g0586600	38.02	D	2.82	U	53.11	D	1.50	U
25	GNAT	Os03g0205800	5.79	U	10.28	U	5.43	U	6.77	U
26	MADS	Os12g0501700	29.97	U	86.44	U	5.79	U	12.58	U
27	MYB-related	Os05g0579600	52.39	D	28.25	D	7.56	D	13.03	D
28	MYB-related	Os06g0728700	9.13	D	6.10	U	16.15	D	3.19	U
29	NAC	Os12g0477400	7.13	U	22.52	U	2.00	U	8.33	U
30	NAC	Os11g0126900	3.56	U	11.59	U	1.06	D	6.55	U
31	HB	Os03g0109400	5.61	U	17.60	U	3.09	U	7.98	U
32	HMG	Os02g0258200	1.02	U	7.29	D	14.23	D	7.63	D
33	MYB-related	Os02g0685200	2.34	D	10.57	U	5.75	D	3.37	U
34	PHD	Os06g0187000	1.10	U	6.72	D	22.53	D	3.62	D
35	GRAS	Os06g0127800	1.14	U	3.59	D	30.11	D	7.77	D
36	GRF	Os02g0678800	1.39	U	1.75	D	11.22	D	2.60	D
37	HB	Os10g0575600	1.78	U	1.62	D	12.97	D	1.25	D

* T_0_ and R_0_ refer to rice cultivars TN1 and RHT before attack by BPH (0 h); T_8_, T_24_, R_8_, and R_24_ refer to the same cultivars sampled 8 or 24 h after attack by BPH; FC, fold change; RP, regulation pattern; U, up-regulation; D, down-regulation.

### Transcription factor genes most likely related to constitutive resistance in RHT

To identify some specific TF genes from the resistant rice variety RHT, we analyzed TF gene expression in RHT uninfested or infested with BPH. In total, 26 genes had FC values of >10 (*P*<0.05) (Table 2). These TF genes were divided into three categories: (1) Those that were strongly up- or down-regulated in RHT compared with TN1 before BPH infestation (13 genes, Table 2, lines 1–13). The expression patterns of these genes showed little difference before and after BPH infestation; therefore, these were genes that were constitutively expressed at high levels during the normal life cycle, and were not likely to be involved in the BPH resistance response. (2) Those that were strongly up- or down-regulated in RHT compared with TN1 before BPH attack (5 genes, Table 2, lines 14–19). The expression levels of these genes changed significantly after BPH attack. Therefore, these genes were probably involved in BPH resistance by regulating expression of resistance-related genes. (3) Those that showed almost the same expression levels in TN1 and RHT before BPH attack, but changed significantly in RHT after BPH attack. The remaining eight TF genes were in this category. These were the TF genes that most probably participated in BPH resistance in RHT (Table 2, lines 19–26).

**Table 2:** **TF genes that were unaffected by BPH and those that showed constitutive differential expression in RHT (FC>10, *P*<0.05)**

**No**	**Family**	**Gene ID**	**R**_**0**_**/T**_**0**_		**R**_**8**_**/T**_**8**_		**R**_**24**_**/T**_**24**_	
			**FC**	**RP**	**FC**	**RP**	**FC**	**RP**
1	bHLH	Os01g0108400	60.02	U	30.02	U	59.83	U
2	MYB-related	Os07g0634900	584.50	U	1219.35	U	216.66	U
3	C3H	Os01g0192000	24.32	U	25.73	U	25.62	U
4	FAR1	Os05g0128700	20.44	D	106.39	D	29.94	D
5	FAR1	Os04g0316800	10.96	D	12.35	D	17.78	D
6	GNAT	Os08g0260000	9.35	D	9.66	D	12.28	D
7	MADS	Os08g0112700	9.61	U	37.64	U	23.08	U
8	MADS	Os04g0387500	12.88	D	41.15	D	13.56	D
9	mTERF	Os06g0224400	7.50	D	6.11	D	23.84	D
10	GNAT	Os02g0806000	14.71	U	21.75	U	19.65	U
11	Orphans	Os01g0530300	6.53	U	5.20	U	50.91	U
12	TRAF	Os03g0667100	223.89	D	118.10	D	110.88	D
13	TRAF	Os08g0227200	21.53	U	33.62	U	17.75	U
14	bHLH	Os04g0301500	5.53	D	13.20	D	2.85	D
15	bZIP	Os01g0859500	10.39	D	6.31	D	1.17	U
16	FAR1	Os08g0389800	25.22	U	4.59	U	1.56	U
17	FAR1	Os06g0246700	10.18	D	1.50	U	1.35	U
18	Orphans	LOC_Os04g13480.1	25.93	U	1.16	D	3.73	U
19	Tify	Os09g0439200	4.82	U	7.20	U	30.32	U
20	TRAF	LOC_Os08g41240.1	4.41	D	3.97	D	10.30	D
21	MYB	Os02g0624300	2.17	D	11.48	D	4.38	D
22	ARF	Os04g0664400	1.99	U	14.99	D	4.05	U
23	NAC	Os01g0339500	1.70	D	1.64	U	10.83	D
24	SET	Os08g0244400	1.92	U	18.66	U	8.11	U
25	TCP	Os03g0706500	1.36	D	11.25	D	2.18	U
26	Tify	Os04g0395800	1.10	U	14.08	D	1.20	D

* T_0_ and R_0_ refer to rice cultivars TN1 and RHT before attack by BPH (0 h); T_8_, T_24_, R_8_, and R_24_ refer to the same cultivars sampled 8 or 24 h after attack by BPH; FC, fold change; RP, regulation pattern; U, up-regulation; D, down-regulation.

### Confirmation of microarray expression profiles by qPCR

**Figure 4:**
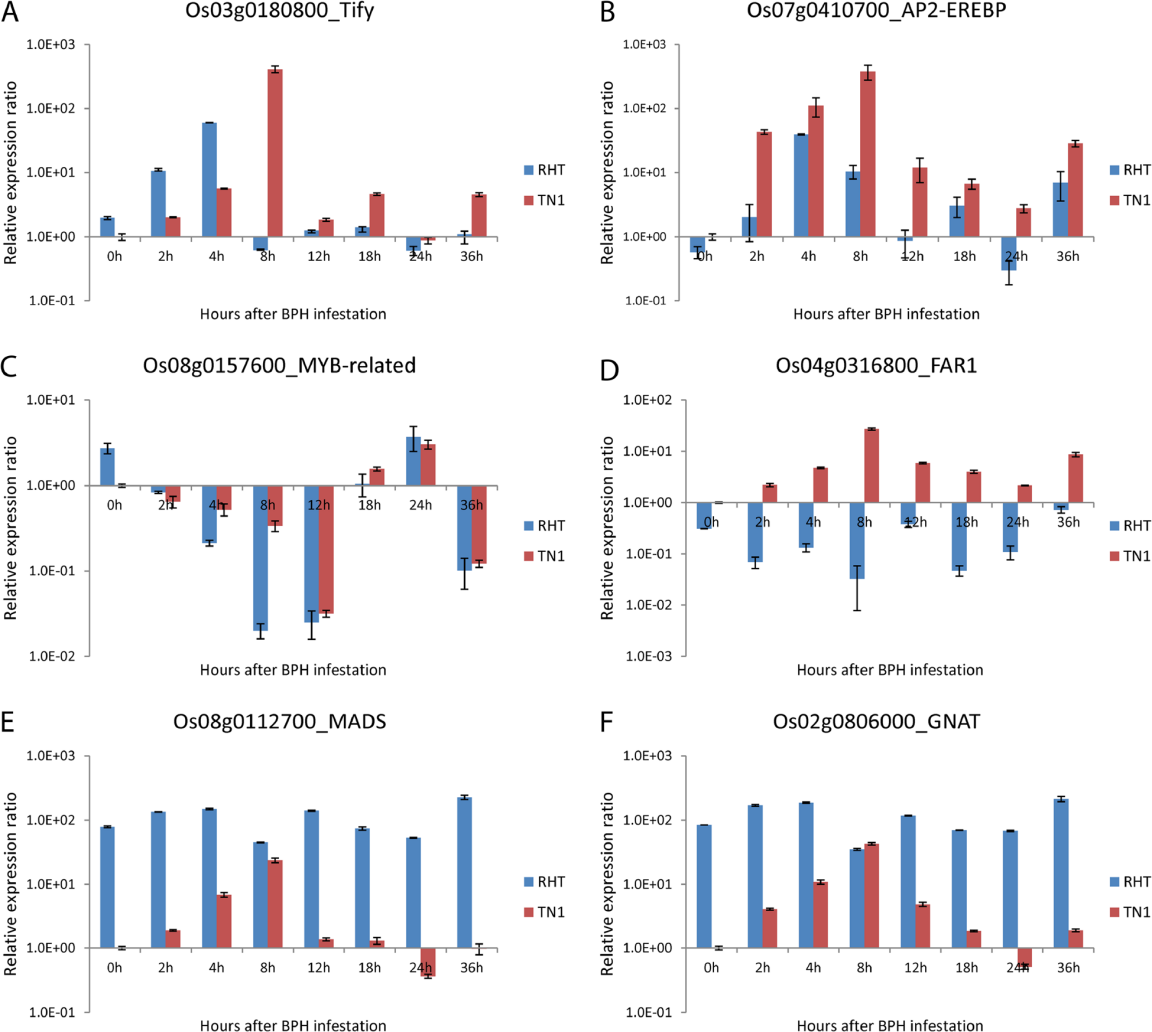
**Results of qRT-PCR analyses to verify TF gene expression profiles in RHT and TN1 at different time-points before and after BPH attack.A**, **B** and **C**: Three TF genes related to BPH-induced resistance. **D**, **E** and **F**: Three TF genes related to constitutive BPH resistance in RHT.

To verify the expression profiles of TF genes, we selected six TF genes for qPCR analyses, and increased the number of analyzed time points (8 in total). The expression patterns of all six TF genes determined by qPCR were the same as those predicted from microarray analyses (Figures 4 A–F). Figures 4A, 4B, and 4C show expressions of three BPH-induced TF genes. Figure 4A shows expression of a Tify family TF gene; its expression was up-regulated in RHT and TN1 after BPH infestation. When this result is compared with data in Table 1 (line 13, showing expression at 8 and 24 h after BPH infestation), a slight difference was that its expression was up-regulated at 4 h after BPH infestation. Therefore, for accurate analysis of expressions of genes induced by BPH, qPCR analyses provided additional data to array data. Figures 4D, 4E, and 4F illustrate expressions of three TF genes showing differential expression between uninfested and infested RHT. In the resistant variety RHT, these genes were up- or down-regulated before and after BPH infestation (Figure 4 D–F, blue bars). However, in the susceptible rice variety TN1, their expressions were up-regulated after BPH attack (Figure 4 D–F, red bars). These TF genes represent those that are constitutively expressed in RHT in the normal life cycle, and most are unlikely to be involved in the BPH resistance reaction.

## Discussion

Studies on the resistance mechanism of rice to BPH have been based on physical interactions, and more recently, on gene expression levels [9,11,46-48]. There is increasing evidence that TF genes play important roles in plant defense responses against phloem-feeding insects. TF genes represent a key element in the modification of gene expression during the plant defense reaction [8,17,18,49].

Here, we provide an overview of the expression profile of TF genes related to BPH resistance, based on comparisons of susceptible and resistant rice plants. Using TF gene probes, we detected 2038 TF genes showing differential expressions between the two rice varieties RHT and TN1 (Additional file 2). Of these, 442 TF genes were probably related to BPH-induced resistance, because their expressions were increased or decreased in response to BPH infestation (Additional file 3). All these induced TF genes showed an interesting expression pattern at two time points (8 or 24 h) after BPH infestation (Figure 2A, 2B). After BPH feeding, the susceptible strain TN1 had more up- or down-regulated TF genes than did the resistant strain RHT. According to the different patterns of gene expression profiles, more TF genes were induced by BPH in the susceptible rice variety TN1 than in the resistant rice variety RHT. For instance, as shown in Figure 2A and 2B, TF genes specially up-regulated at 8, and 24 hours in TN1 were 105 and 154, respectively; while the numbers in RHT were 28 and 31. And TF genes specially down-regulated at 8, and 24 hours in TN1 were 61 and 65, respectively; while the numbers in RHT were 39 and 20. Because induced resistance is defined from the point of view of the herbivore and it does not necessarily benefit the plant. This means that the induced resistance may render the plant more susceptible to other stresses [4]. This may be the one important reason why TN1 rice is more susceptible than RHT. Further analysis results also support this point of view. In TN1, there were more up-regulated than down-regulated TF genes in response to BPH. In contrast, in RHT, there were more down-regulated than up-regulated genes in response to BPH (Figures 2A). This finding suggested that the damage caused by BPH feeding to the susceptible variety TN1 triggered expressions of a series of genes. The products of these genes may play roles in repairing damage to the phloem to prevent the loss of phloem sap, and in defense against invasion of the pathogen and bacteria. In the resistant variety RHT, this reaction involved cessation of several metabolic pathways to prevent the loss of phloem sap, and metabolic activity was repressed throughout the entire plant. This is an efficient method of defense against BPH feeding, because it reduces the amount of substances supplied to the phloem, similar to the function of the resistance gene *Bph14*[49]. Therefore, various TFs could have different functions in this reaction. This finding provided evidence at the molecular level for why the resistant cultivar, RHT, suffered less damage than the susceptible cultivar, TN1.

Studies on the physical test phenotype by EPG (electrical penetration graph) of these cultivars showed that TN1 had a greater damage score after exposure to BPH, while the phloem sap of RHT could not be extracted constantly by BPH stylets [1,45]. According to this study, the expressions of many TF genes were induced in TN1 by exposure to BPH. This would probably lead to metabolic disorders in TN1, which probably the reason why this rice variety is susceptible to BPH. However, in RHT, far fewer TF genes were induced after BPH exposure. Most of the TF genes showing altered expressions in RHT were down-regulated (Figure 2A and 2B), so we boldly deduced that certain metabolic pathways were turned off. This may explain why BPH was unable to extract nutrition from the RHT phloem sap.

Among the down-regulated TF genes, all members of FHA, Orphans, and Sigma70-like families, and most members of the MYB family were significantly down-regulated at 8 h after BPH infestation (Additional file 5). A similar pattern of regulation also occurred in *Arabidopsis* after aphid infestation [17]. Members of the MYB family have highly diversified biological functions, and the expressions of most *Arabidopsis* MYB genes were responsive to one or multiple types of hormone and stress treatments [50]. Studies of the *Arabidopsis* defense response to chewing insects showed that knockdown of a MYB gene, *AtMYB102*, enhanced susceptibility to white cabbage butterfly (*Pieris rapae*) [51]. In tobacco, transgenic plants overexpressing a wheat MYB gene, *TaPIMP1*, showed significantly enhanced resistance to the pathogen *Ralstonia solanacearum*, and increased tolerance to drought and salt stresses [30]. Another study on TFs suggested that several members of the MYB family play important roles in photosynthesis and metabolism [52]. During their physiological responses to insects, plants reallocate energy from photosynthesis to the defense response [9]. During BPH feeding, the chlorophyll level, net photosynthetic rate, stomatal conductance, and transpiration rates significantly decreased in the susceptible cultivar, MH63 [11]. These patterns of decreased metabolic activity were consistent with the down-regulation of MYB family genes observed in BPH-infested rice plants in this study. The down-regulation of these genes may have played a role in reducing photosynthesis. Microarray analyses have shown that down-regulation of TF genes occurs during compatible plant-aphid interactions [53].

In both rice varieties used in the present study, the up-regulated TF genes after 8-h of BPH infestation included most members of the AP2-EREBP, NAC, and WRKY families and all members of the ABI3VP1 family (Additional file 5). The AP2 TF family is one of several that are unique to the plant lineage. This family, whose members contain an EREBP (ethylene responsive element binding factor) domain, is involved in regulation of plant disease resistance [19]. Genes encoding members of the AP2-EREBP family of TFs are also involved in plant resistance to insects. The AP2-EREBP TF response to herbivore attack might depend on a hormone-dependent signaling pathway. Over-expression of *OsERF3* positively regulated TrypPI activity and boosted the resistance of rice to the chewing herbivore SSB, whereas it negatively regulated resistance against BPH [8]. The expression pattern of up-regulated AP2-EREBP genes may reflect those induced by physical damage during BPH feeding, and the down-regulated AP2 TFs may be involved in the BPH resistance interaction. Previous studies on NAC, WRKY, and Zn-finger TF families mainly focused on responses to fungal and bacterial pathogens [23,25,33]. According to those studies, their functions rely on JA and SA signaling pathways, which are involved in the BPH-resistance response.

We narrowed down the number of TF genes that were probably involved in BPH resistance by increasing the FC value to >10 (*P*<0.05). At this FC level, there were 37 TF genes that were BPH-induced and 26 associated with constitutive resistance in RHT. Further analysis indicated that there were 13 and 8 TF genes most probably involved in BPH-induced and constitutive resistance, respectively. Further research is underway to study these 21 genes in more detail. Most of the microarray expression profiles were consistent with the qPCR results. (Figure 4 & Additional file 7). However, only a few time points were analyzed in the microarray analyses, and so it is difficult to determine the exact time at which gene expression peaked after induction by BPH. To address this point, more time points should be included in qPCR analyses of gene expression.

## Conclusions

TFs are up-stream regulators that control transcription by DNA binding. By analyzing the TF gene expression profiles after BPH feeding, especially those of genes that were differentially expressed between resistant (RHT) and susceptible (TN1) rice varieties, we obtained information about the resistance mechanism to BPH at the molecular level. Our results indicate that the levels of gene expression play an important role in the plant defense reaction. The down-regulation of TF genes will repress many active pathways, which can prevent further damage related to loss of water and nutrients.

An interesting aspect of the molecular mechanism of TFs in plant resistance to BPH was that the TF genes showing down-regulated or repressed expressions were probably the main reason for BPH resistance in RHT. We identified a total of 21 TF genes that are probably involved in BPH resistance in the resistant rice variety, RHT.

## Abbreviations

BPH: Brown planthopper; TF: Transcription factors; RHT: Rathu Heenati; TN1: Taichun Native 1; FC: Fold change; HR: Hypersensitive response; SA: Salicylic acid; JA: Jasmonic acid; ET: Ethylene; WBPH: White-backed planthopper; qPCR: Quantitative reverse-transcription PCR.

## Competing interests

The authors declare that they have no competing interests.

## Authors’ contributions

YW, XM conceived and designed the study, and wrote the manuscript. YW and HL analyzed the experiment results, prepared figures and tables, and revised the manuscript. HG and HZ collected samples and conducted qRT-PCR analyses. All authors read and approved the final manuscript.

## Supplementary Material

Additional file 1 Primer pairs for quantitative RT-PCR to verify relative expression levels of transcription factor genes. Click here for file

Additional file 2 **All transcription factor genes detected by microarray analyses.** This file lists all 2038 transcription factor genes detected by microarray analyses, and shows TF genes showing differential expression profiles between two rice varieties TN1 and RHT before and after attack by BPH. Click here for file

Additional file 3 **Transcription factor genes related to BPH-induced resistance showing changes in expression with fold-change values of >2 after BPH infestation (*P*<0.05).** This file lists 442 transcription factor genes showing changes in expression after BPH attack (fold-change >2, *P*<0.05). It also shows TF genes showing differential expression profiles between two rice varieties TN1 and RHT before and after attack by BPH. Click here for file

Additional file 4 **Transcription factor genes related to constitutive resistance in RHT showing changes in expression with fold-change values of >2 (*P*<0.05).** This file lists 229 transcription factor genes that were up-regulated or down-regulated before and after BPH attack in RHT compared with their respective expressions in TN1. Click here for file

Additional file 5 **BPH-induced transcription factor genes showing altered expression after BPH infestation with fold-change values of >5 (*P*<0.05).** This file lists 119 transcription factor genes showing altered expression after BPH attack with fold-change values of >5 (*P*<0.05). It also shows TF genes showing differential expression profiles between two rice varieties of TN1 and RHT before and after attack by BPH. Click here for file

Additional file 6 **Transcription factor genes related to constitutive resistance in RHT with fold-change values of >5 (*P*<0.05).** This file lists 66 transcription factor genes showing up- or down-regulated expressions before and after BPH attack in RHT compared with their respective expressions in TN1. Click here for file

Additional file 7 The other results of qRT-PCR analyses to verify TF gene expression profiles in RHT and TN1 at different time-points before and after BPH attack. Click here for file
